# Bactericidal Activities of Nanoemulsion Containing *Piper betle* L. Leaf and Hydroxychavicol Against Avian Pathogenic *Escherichia coli* and Modelling Simulation of Hydroxychavicol Against Bacterial Cell Division Proteins

**DOI:** 10.3390/antibiotics14080788

**Published:** 2025-08-03

**Authors:** Kunchaphorn Ratchasong, Phirabhat Saengsawang, Gorawit Yusakul, Fonthip Makkliang, Hemanth Kumar Lakhanapuram, Phitchayapak Wintachai, Thotsapol Thomrongsuwannakij, Ozioma Forstinus Nwabor, Veerasak Punyapornwithaya, Chonticha Romyasamit, Watcharapong Mitsuwan

**Affiliations:** 1 Program in Agriculture and Food Science, College of Graduate Studies, Walailak University, Nakhon Si Thammarat 80160, Thailand; kunchaphorn.ra@mail.wu.ac.th (K.R.); hemanthkumar.la@st.wu.ac.th (H.K.L.); 2 Akkhraratchakumari Veterinary College, Walailak University, Nakhon Si Thammarat 80160, Thailand; phirabhat.sa@wu.ac.th (P.S.); thotsapol.th@wu.ac.th (T.T.); 3 One Health Research Center, Walailak University, Nakhon Si Thammarat 80160, Thailand; 4 Department of Pharmaceutical Chemistry and Pharmacognosy, Faculty of Pharmaceutical Sciences, Naresuan University, Phitsanulok 65000, Thailand; gorawity@nu.ac.th; 5 School of Languages and General Education, Walailak University, Thasala, Nakhon Si Thammarat 80160, Thailand; fonthip.ma@wu.ac.th; 6 Functional Materials and Nanotechnology Center of Excellence, School of Sciences, Walailak University, Thasala, Nakhon Si Thammarat 80160, Thailand; phitchayapak.wi@wu.ac.th; 7 Department of Public Health, Syracuse University, Syracuse, NY 13244, USA; ofnwabor@syr.edu; 8 Research Center for Veterinary Biosciences and Veterinary Public Health, Faculty of Veterinary Medicine, Chiang Mai University, Chiang Mai 50100, Thailand; veerasak.p@cmu.ac.th; 9 School of Allied Health Sciences, Walailak University, Nakhon Si Thammarat 80160, Thailand; chonticha.ro@wu.ac.th; 10 Center of Excellence in Innovation of Essential Oil and Bio-Active Compound, Walailak University, Nakhon Si Thammarat 80160, Thailand

**Keywords:** avian pathogenic *Escherichia coli*, *Piper betle*, nanoemulsion, hydroxychavicol, antibacterial activities, cell division proteins

## Abstract

**Background**: Avian pathogenic *Escherichia coli* (APEC) is a leading cause of colibacillosis in poultry. *Piper betle* L. is a medicinal plant rich in bioactive compounds including hydroxychavicol that possess potent antibacterial activity. This study aimed to investigate the efficacy of a *P. betle* L. leaf nanoemulsion (NEPE) and hydroxychavicol against multidrug-resistant APEC isolates. **Methods**: In vitro and in silico analysis of NEPE and hydroxychavicol against APEC were determined. **Results**: The nanoemulsion exhibited potent antibacterial activity, with MIC and MBC values of 0.06–0.25% *v/v* and 0.125–0.25% *v/v*, respectively. The MIC and MBC values of hydroxychavicol against isolates ranged from 0.25 to 1.0 mg/mL. A time–kill assays revealed rapid bactericidal effects of both compounds, achieving a ≥3-log reduction within 4 h at 4 × MIC. Scanning electron microscopy demonstrated that APEC cells treated with hydroxychavicol exhibited filamentous cells with incomplete septa. Molecular docking and dynamics simulations of hydroxychavicol against APEC cell division proteins were investigated. According to the binding energy, hydroxychavicol exhibited the highest affinity with ZapE, FtsW, FtsX, FtsZ, and FtsA, respectively. However, the FtsA protein showed the least protein conformational change throughout the 5000 ns simulation, reflecting a highly stable conformation. **Conclusions**: These confirm the potential stability of protein and ligand, as supported by molecular dynamics simulation. The results suggested the potential of NEPE and hydroxychavicol, which may have promising antibacterial potential that can be used to inhibit APEC growth.

## 1. Introduction

Avian pathogenic *Escherichia coli* (APEC) is a major cause of colibacillosis in poultry, a serious disease that leads to high mortality rates in chickens and economic losses in the poultry industry. As this bacterium belongs to the class of facultative anaerobic organisms and is resistant to most environmental stressors, newly hatched chickens are likely to come into contact with *E. coli*. In newly hatched chickens, *E. coli* infections may be initiated as navel and yolk sac infections associated with septicemia. Most chicken mortality caused by APEC occurs within the first week of infection [1]. APEC strains are characterized by their ability to cause various diseases, including respiratory infections and septicemia. APEC can also be zoonotic [2]. APEC has several important pathogenic mechanisms, such as cell surface adhesion, cell invasion, and biofilm formation. These factors may result in increased pathogenicity, making the treatment of infections caused by such organisms difficult [3]. One of the growing challenges in managing APEC infections is increasing antibiotic resistance.

Alternative therapeutic strategies targeting bacterial cell division machinery, such as filamentous temperature-sensitive protein Z (FtsZ), have gained prominence as a promising approach to combat antimicrobial resistance [4]. Filamenting temperature-sensitive mutant Z (FtsZ), a eukaryotic tubulin homolog, is a GTP-dependent prokaryotic cytoskeletal protein and is conserved among most bacterial strains [5]. In *E. coli*, cell division is orchestrated by the divisome, a dynamic multiprotein complex where FtsZ serves as the principal scaffold. The process initiates with GTP binding, which triggers FtsZ polymerization into protofilaments that coalesce into a circumferential Z-ring at the middle of the cell. This structure is stabilized through interactions with membrane-anchoring proteins such as ZipA and FtsA, ensuring proper spatial localization. Subsequent divisome maturation involves the sequential recruitment of auxiliary proteins, including FtsI (penicillin-binding protein 3), which facilitates septal peptidoglycan biosynthesis. Ultimately, coordinated constriction of the Z-ring, membrane invagination, and cell wall remodeling lead to daughter cell separation. The indispensability of FtsZ arises from its dual role as a GTPase-driven structural organizer and a regulatory hub for divisome assembly, making it a compelling target for antimicrobial intervention. Molecular docking and simulation are computational techniques of modern drug discovery, more so in cases where the targeted proteins are critical for the bacterial cell division process, such as FtsZ. These computational techniques occupy a central place in understanding molecular interactions between potential drug candidates [6]. Hence, they offer several advantages that make them indispensable in the early stages of drug development.

*Piper betle* L. is a species of flowering plant in the Piperaceae family and is widely recognized as a common medicinal plant in Southeast Asia. The leaves, known for their diverse bioactive compounds, are the most extensively studied part due to their antimicrobial, antioxidant, and anti-inflammatory properties [7]. *P. betle* leaves exhibited very high efficacy against Gram-negative bacteria, especially against *E. coli* [8], due to their bioactive compounds, including hydroxychavicol and eugenol. These components possess strong antifungal and antibacterial properties [9], which has garnered significant attention from researchers for their pharmacological and biological activities [10]. *P. betle* extract was shown to exhibit antimicrobial activity by disrupting microbial cell membranes, inhibiting microbial growth, and preventing biofilm formation [11]. The main limitation of *P. betle* ethanol extract lies in its poor water solubility. Because the extract contains hydrophobic bioactive compounds such as hydroxychavicol, it does not readily dissolve in aqueous environments, leading to challenges in achieving uniform dispersion and bioavailability.

As an independent lipid-based drug delivery technology, nanoemulsions have been shown to be quite effective in incorporating hydrophobic active pharmaceutical ingredients into their internal oily phase for protection against degradation [12], highlighting their low toxicity, tunable solvency, biodegradability, and suitability as green solvents for enhancing the solubility and stability of poorly soluble drugs [13]. Nanoemulsions are thermodynamically unstable systems that necessitate external energy inputs, such as high-shear mixing or ultrasonication, for their formation [14]. In contrast, microemulsions are thermodynamically stable systems [14]. Both systems typically consist of an oil phase, an aqueous phase, and a surfactant or co-surfactant, and both construct small droplets, generally less than 100 nm in size [14]. However, microemulsions usually exhibit even smaller, more uniform droplets and can display a variety of structures, whereas nanoemulsions predominantly feature spherical droplets [14]. Hydrophobic deep eutectic solvents (HDESs), a subclass of deep eutectic solvents (DESs) typically composed of two or more hydrophobic components, can be used as the oil phase of an emulsion instead of conventional and synthetic oils. HDESs can extract hydrophobic compounds and can serve as an active substance against microorganisms [15,16,17]. HDES-based emulsion systems significantly improve the potential of the antimicrobial drug delivery platform with enhanced bioavailability and stability [18,19]. Nanoemulsions have demonstrated significant utility as carriers for antimicrobial, therapeutic, and other bioactive agents, broadening their applications in pharmaceutical, food, and veterinary fields. Notably, recent studies have shown that HDES components, such as menthol and decanoic acid, effectively solubilize hydrophobic drugs and can be formulated into nanoemulsions with nanoscale droplet sizes, leading to substantial improvements in drug solubility and bioavailability [20]. This combination of HDESs and nanoemulsion technology presents a versatile platform for controlled drug release and enhanced therapeutic outcomes. They exhibit some advantages, such as thermodynamic stability, large solubilizing capacity, and isotropy, that are apt for pharmaceutical and cosmetic use. Based on these features and their potential for improving the efficiency of drug loading and bioavailability, nanoemulsions have been found to be quite useful for delivering bioactive compounds from *P. betle*.

This study aimed to investigate the antibacterial activity of nanoemulsion containing *P. betle* leaf (NEPE) and hydroxychavicol against clinical APEC isolates. The determination of MIC and MBC values of NEPE and hydroxychavicol on the clinical isolates of the pathogens, time–kill study, and morphology of the bacteria after treatment with NEPE and hydroxychavicol were investigated. Molecular docking and dynamic simulation were further determined.

## 2. Results

### 2.1. Formulations of the NEPE and Detection of Hydroxychavicol in NEPE

**Figure 1:**
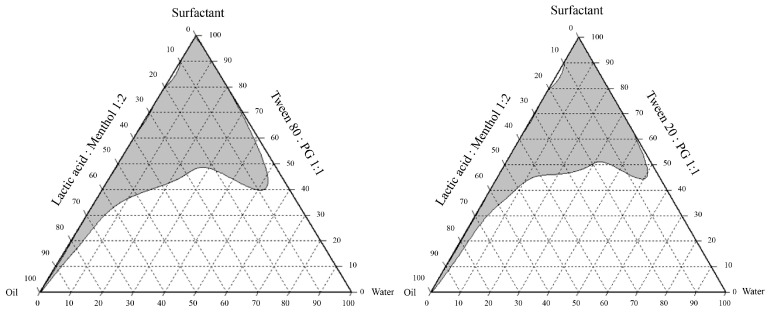
Pseudo-ternary phase diagram analysis of oil–water–surfactant systems: lactic acid:methanol (1:2) with Tween 80:PG and Tween 20:PG (1:1).

**Figure 2:**
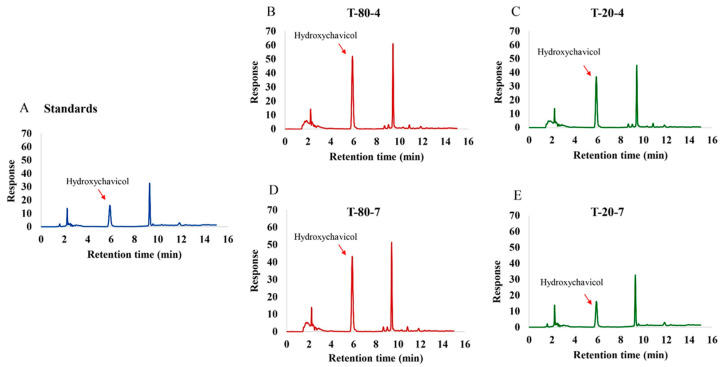
Detection of hydroxychavicol in different formulations of the NEPE (**B**–**E**), and chromatogram of the reference standard (**A**), with commercial hydroxychavicol used for peak identification in the sample formulations.

This study evaluated four different nanoemulsion formulations containing *P. betle* (Table 1), maintaining constant water (10 g) and *P. betle* powder (10 g) across all formulations. The key variables tested were the type of surfactant (Tween 80 and Tween 20) and the ratio of lactic acid and menthol to the surfactant–co-surfactant mixture (Tween: Propylene glycol). The droplet size, polydispersity index (PDI), and zeta potential of various NEPE formulations are summarized in Table 2. Among the tested formulations, T-80-4 exhibited the smallest mean droplet size (162.600 ± 4.255 nm), followed by T-80-7 (353.300 ± 26.535 nm), while T-20-4 and T-20-7 showed significantly larger sizes (642.333 ± 121.643 nm and 24,693.333 ± 899.790 nm, respectively). The PDI values of T-80-7 (0.313 ± 0.044) and T-80-4 (0.459 ± 0.009) indicated relatively narrow droplet size distributions, whereas the higher PDI values observed in T-20-4 (0.622 ± 0.082) and T-20-7 (0.607 ± 0.091) suggested broad distributions and heterogeneity. In terms of zeta potential, T-80-7 and T-80-4 displayed the highest negative charges (−47.633 ± 0.094 mV and −45.533 ± 0.170 mV, respectively), implying good electrostatic stability. In contrast, T-20-4 and T-20-7 exhibited lower absolute zeta potential values (−23.967 ± 0.806 mV and −16.033 ± 0.531 mV), which may lead to reduced colloidal stability. The pseudo-ternary phase indicates that the nanoemulsion region is larger and more extensive in the system using Tween 80:PG (1:1) compared to Tween 20:PG (1:1) when combined with a lactic acid:menthol (1:2) oil phase (Figure 1). Hydroxychavicol, a bioactive compound in *P. betle* leaves, was quantified in the NEPE using HPLC, where the peak area directly correlated with its concentration (Figure 2A–E). Among the four NEPE formulations analyzed, formulation T-80-4 exhibited the highest hydroxychavicol content (4.241 ± 0.010 mg/mL) (Figure 2B) compared to the standard (Figure 2A), followed by T-80-7 (2.624 ± 0.05 mg/mL) (Figure 2D), T-20-4 (2.195 ± 0.009 mg/mL) (Figure 2C), and T-20-7 (1.880 ± 0.005 mg/mL) (Figure 2E), respectively. NEPE formulations with T-80-4 exhibited hydroxychavicol levels of 2.65, 5.30, and 10.6 µg/mL at concentrations of 0.06%, 0.125%, and 0.25% *v/v*, respectively. T-80-7 showed hydroxychavicol contents of 1.64, 3.28, and 6.56 µg/mL. Formulations with T-20-4 contained 1.37, 2.74, and 5.49 µg/mL, while those with T-20-7 exhibited the lowest levels of 1.18, 2.35, and 4.70 µg/mL, respectively.

### 2.2. Antibacterial Activity of NEPE and Hydroxychavicol Against APEC

Antibacterial activity of NEPE formulations and hydroxychavicol against ten clinical isolates of APEC was determined by broth microdilution assay. All NEPE formulations showed similar MIC values ranging from 0.06 to 0.25 *v/v* (Table 3). The MBC values of all NEPE formulations against isolates were 0.125–0.25 *v/v*. It was observed that the MIC of NEPE-T-80-4 was lower than that of other formulations, but it was not significant. Hydroxychavicol possessed strong antibacterial activity against the clinical isolates with identical MIC and MBC values of 0.25 mg/mL. In addition, the MIC and MBC values of the ethanol extract of *P. betle* leaves against the isolates were 1.0 mg/mL. The MIC and MBC values of the antibiotic against the isolates are presented in Table 3. In addition, the MIC and MBC values of the base nanoemulsion without *P. betle* were also tested against the isolates. It was found that the MIC and MBC values of the nanoemulsion base solution were approximately 10 times higher in the nanoemulsion containing *P. betle* (Table S1). 

### 2.3. Time–Kill Study 

**Figure 3:**
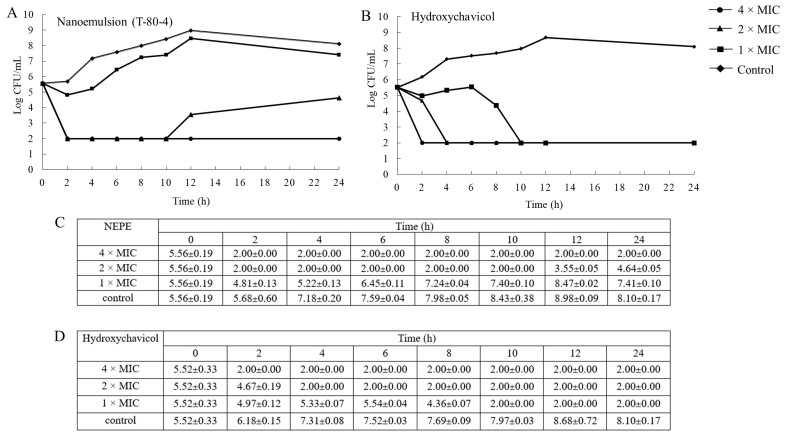
Time–kill curves (**A**,**B**) and CFU Log reduction in the time–kill assay (**C**,**D**) of NEPE (**A**,**C**) and hydroxychavicol (**B**,**D**) against APEC (CHUL50).

A time–kill curve was determined to confirm the antibacterial efficacy of NEPE (T-80-4) and hydroxychavicol against a representative isolate of APEC (Figure 3A–D). The bacterium was treated with the extract at 4 × MIC, 2 × MIC, and 1 × MIC of NEPE and hydroxychavicol. One percent of DMSO was used as a negative control. The results indicated that the antibacterial activity of the NEPE and hydroxychavicol was concentration-dependent due to the inhibition of bacterial growth. A reduction in 3-log bacterial viability was observed after the treatment by the NEPE and hydroxychavicol at 2 and 4 × MICs within 2 h. Furthermore, hydroxychavicol at 1 × MIC showed killing activity against the pathogen within 10 h. 

### 2.4. Viability of APEC After Treatment with NEPE and Hydroxychavicol as Observed by Confocal Microscopy

**Figure 4:**
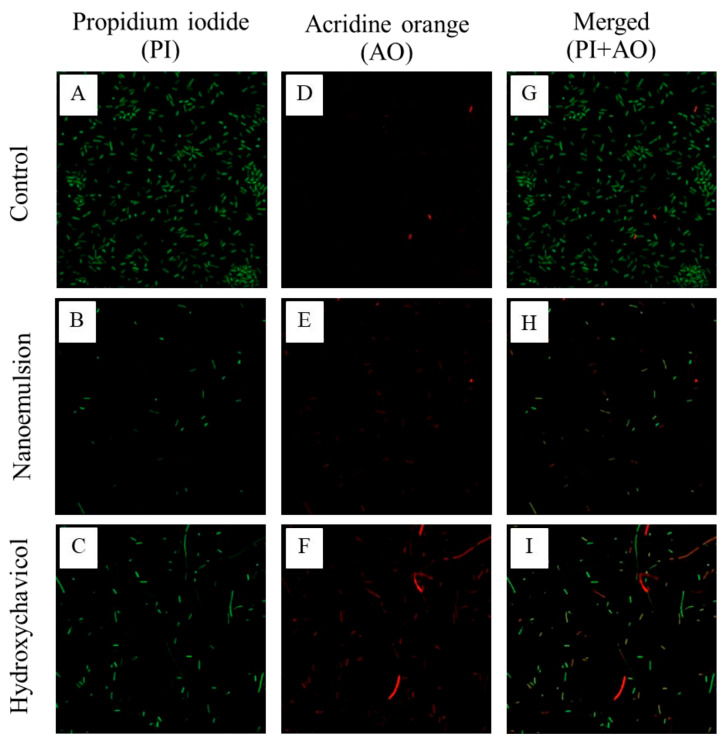
Observation of APEC after treatment with 1 × MIC of NEPE and hydroxychavicol against APEC (CHUL50) (**A**–**I**) by AO/PI viability staining. Live cells fluoresce green, while dead cells fluoresce red.

Confocal microscopy with acridine orange (AO) and propidium iodide (PI) staining is a powerful technique for assessing cell viability and morphology. As shown in Figure 4A–I, rod-shape bacteria with green fluorescence were observed in the nanoemulsion treatment group and untreated control. A long cell shape was observed when APEC cells were treated with hydroxychavicol. In addition, the bacterial cells were green-yellow in color (Merged PI + AO). However, a few bacteria with red fluorescence were observed after treatment with hydroxychavicol. However, no red-stained cells were observed following microemulsion treatment. The authors should explain this observation. 

### 2.5. Morphology of APEC After Treatment with NEPE and Hydroxychavicol by Scanning Electron Microscopy (SEM)

**Figure 5:**
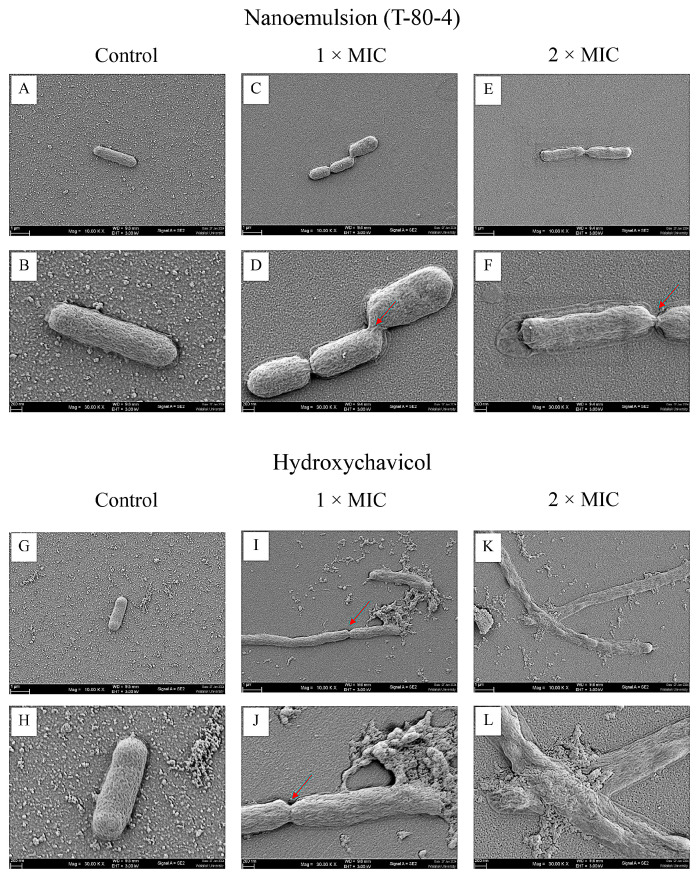
Morphology of APEC (CHUL50) after treatment with the NEPE (**A**–**F**) and hydroxychavicol (**G**–**L**) as observed by SEM. Red arrows indicated division septum.

Scanning electron microscopy (SEM) was used to observe the morphology of APEC cells treated with NEPE and hydroxychavicol. The results showed that bacterial cells in the control group exhibited a typical rod shape with a smooth surface (Figure 5A,B,G,H). However, treatment with hydroxychavicol at 2 × MIC and 4 × MIC resulted in elongated bacterial cells (Figure 5I–L). Notably, some of the treated bacterial cells appeared significantly longer and lacked septa, suggesting potential disruption of cell division. However, a slight effect on the cell elongation after treatment with NEPE was observed (Figure 5C–F).

### 2.6. Molecular Docking and Molecular Dynamics Simulation

**Figure 6:**
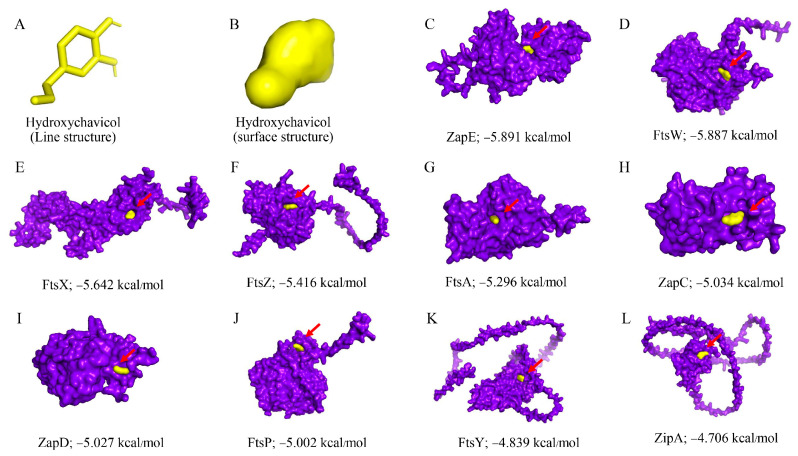
The average binding energy between hydroxychavicol and cell division proteins was calculated. Red arrows indicated the area of hydroxychavicol.

**Figure 7:**
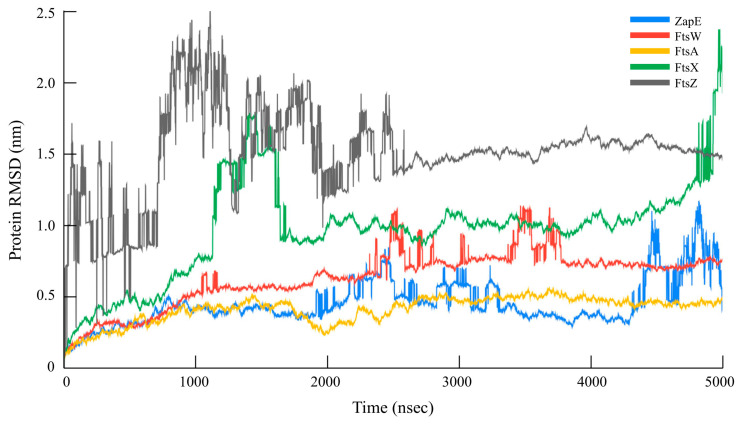
Simulation time of protein RMSD value for cell division proteins with ligand (hydroxychavicol).

**Figure 8:**
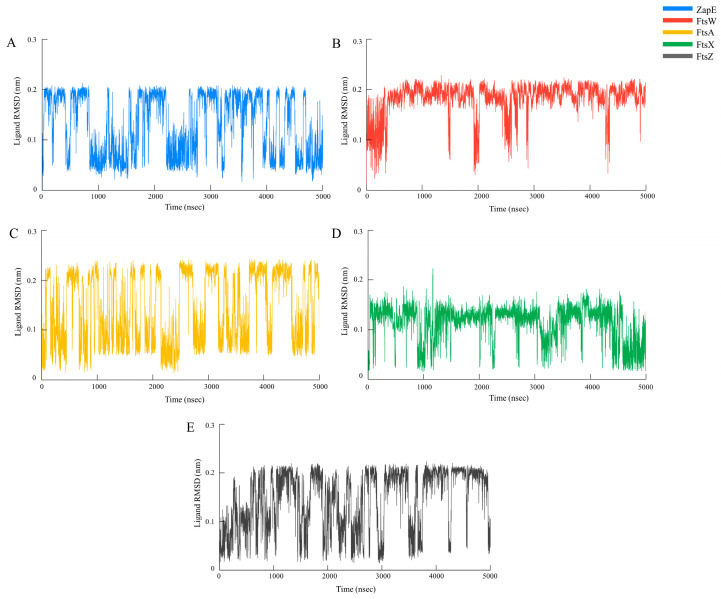
Simulation time of ligand RMSD. The RMSD values for the hydroxychavicol in protein complex with ZapE (**A**), FtsW (**B**), FtsA (**C**), FtsX (**D**), and FtsZ (**E**).

**Figure 9:**
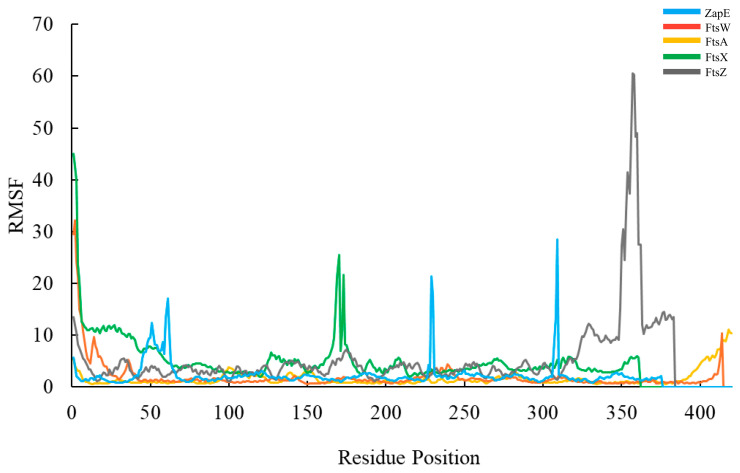
The root mean square fluctuation (RMSF) of simulated proteins.

**Figure 10:**
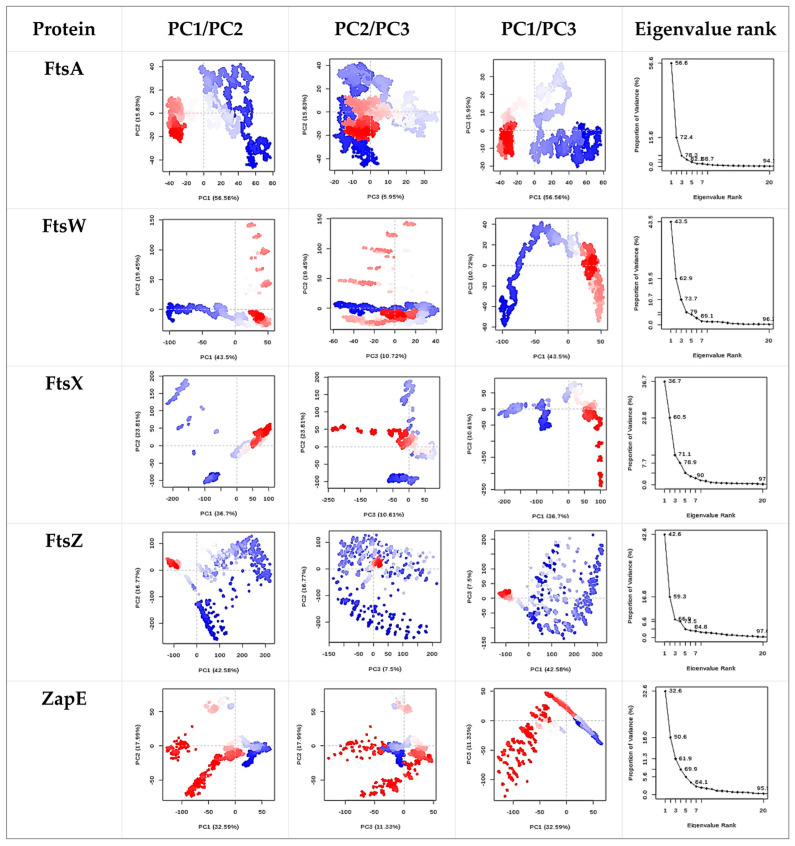
Principal component analysis (PCA) was performed for confirmation of the change in selected proteins that were binding with the hydroxychavicol molecule.

The hydroxychavicol molecule was docked with the cell division protein family, which revealed different binding energies (Figure 6 and Table 4). The average binding energy between hydroxychavicol and the cell division protein family of APEC was −5.274 kcal/mol. Of this, ZapE protein revealed the lowest binding energy (−5.891 kJ/mol), followed by FtsW (−5.887 kJ/mol), FtsX (−5.642 kJ/mol), FtsZ (−5.416 kJ/mol), and FtsA (−5.296 kJ/mol), respectively. These cell division proteins were the top 5 cell division proteins that bound with hydroxychavicol steadily and were selected for further simulation analyses. In addition, the binding energy of the positive control, cefepime, is demonstrated in Table 4. This study focuses on hydroxychavicol interacting with the binding energy of five docked proteins, which were revealed to have a higher chance of molecule interaction. The selected proteins had binding energy less than −5.200 kJ/mol, and we additionally analyzed their molecular dynamic simulations. The root mean square deviation (RMSD) of selected cell division proteins is presented in Figure 7. During 5000 ns, each protein differed in stability when interacting with hydroxychavicol over time. The FtsA (420 amino acids), ZapE (375 amino acids), and FtsW (414 amino acids) were initially structurally stable at around 1000 ns. However, FtsW and ZapE were found to be slightly unstable after 2500 ns. The average RMSD for FtsA, ZapE, and FtsW were 0.41 ± 0.09, 0.45 ± 0.16, and 0.63 ± 0.19 nm, respectively. The FtsA protein showed the least protein conformational change throughout the 5000 ns simulation, reflecting highly stable conformation. For FtsX (361 amino acids) and FtsZ (383 amino acids), proteins were initially stable at around 2500 ns. The average RMSD of the FtsX and FtsZ were 0.96 ± 0.34 and 1.51 ± 0.31 nm, respectively. Meanwhile, FtsX and FtsZ showed high fluctuations, especially FtsZ, indicating high conformational change, which was affected by stability loss upon ligand binding. For the RMSD of hydroxychavicol on each selected protein, the values were approximately changed up to 0.2 nm, which was a very small stability change (Figure 8). FtsW showed the lowest ligand RMSD values (0.18 ± 0.03 nm) and had fewer changes compared to the other proteins, which means that hydroxychavicol remains closely bound to this protein in the binding pocket. In addition, the FtsX revealed lower ligand RMSD values (0.11 ± 0.04 nm) and lower fluctuation than other remaining selected proteins. ZapE, FtsA, and FtsZ indicated that their orientations had quite low stability in the binding interaction. Thus, hydroxychavicol revealed its stable movement within the binding pocket of the proteins throughout the simulation. FtsA revealed the lowest RMSF with a median value of 1.04 ± 0.77, followed by FtsW (1.25 ± 0.67) and ZapE (1.81 ± 0.76), respectively. Conversely, FtsZ (3.54 ± 2.33) and FtsX (4.05 ± 2.36) presented high fluctuations along the entire amino acid positions. The root mean square fluctuation (RMSF) of simulated proteins is presented in Figure 9. In addition, all selected proteins were analyzed with their movements with the top three eigenvalues (56.6–78.3% for FtsA, 43.5–73.7% for FtsW, 36.7–71.1% for FtsX, 42.6–66.9% for FtsZ, and 32.6–61.9% for ZapE). Principal component analysis (PCA) was performed for confirmation of the change in selected proteins that were binding with the hydroxychavicol molecule. All selected proteins revealed minimal variability on PC3 when compared to the other PCs (Figure 10). FtsA presented the lowest variability (5.95%) among the selected proteins, followed by FtsZ (7.50%). For FtsW, FtsX, and ZapE, these proteins showed minimal variability on PC3 similarly (10.61–11.33%). The number of hydrogen bonds between hydroxychavicol and selected proteins was 13 bonds in ZapE, 10 bonds in FtsZ and FtsX, and 4 bonds in FtsZ. However, no hydrogen bonds were found in the complex of FtsW and hydroxychavicol. Of these, molecular dynamic simulations revealed that FtsA was a cell division protein that is most stable when interacting with the hydroxychavicol molecule.

### 2.7. Cell Viability and Nitric Oxide Production

**Figure 11:**
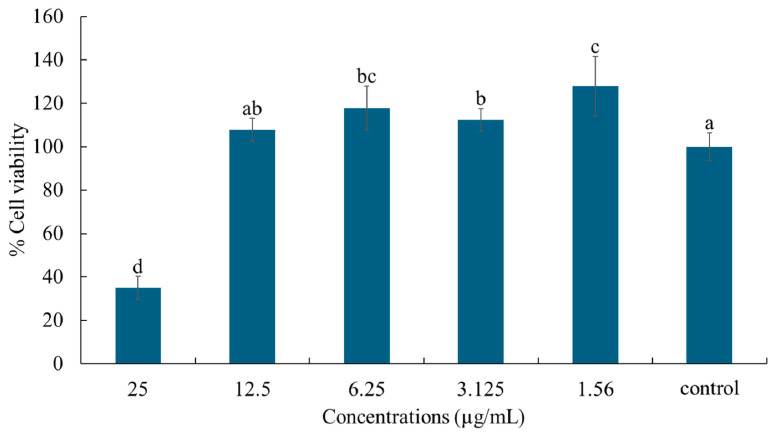
Effects of hydroxychavicol on the viability of RAW 264.7 cells determined by MTT assay. Data are expressed as mean ± standard deviation. Different lowercase letters (a, b, c, d) indicate significant differences among groups (*p* < 0.05) based on Duncan’s test.

The effects of hydroxychavicol on RAW 264.7 cell viability were detected by MTT assay. Hydroxychavicol at 12.5 µg/mL did not cause toxicity to RAW 264.7 cells compared to the control (Figure 11). However, a decrease in cell viability was observed after treatment with hydroxychavicol at 25 µg/mL. To further investigate the effects of hydroxychavicol on nitric oxide production in RAW 264.7 cells, the concentrations of hydroxychavicol that showed a percentage of viable cells >95%(LC5) and viable cells >90%(LC10) were chosen.

**Figure 12:**
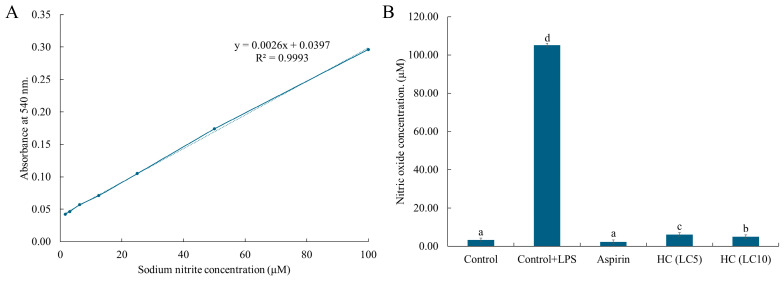
Determination of nitric oxide production in RAW 264.7 cells using Griess assay. The inhibitory activity of hydroxychavicol on nitric oxide production in RAW 264.7 cells induced by LPS was demonstrated (**B**). The level of nitric oxide production was assessed using a standard curve performed by sodium nitrite (**A**). Different lowercase letters (a, b, c, d) indicate significant differences among groups (*p* < 0.05) based on Duncan’s test.

The anti-inflammatory activity of hydroxychavicol against RAW 264.7 cells induced by LPS was investigated by detecting nitric oxide production. A standard curve was prepared using sodium nitrite as a standard compound. A linear equation of the standard curve (y = 0.0026x + 0.0397, R^2^ = 0.9993) was presented in Figure 12A. The results demonstrated that RAW 264.7 cells induced by LPS showed a high level of nitric oxide production with 105.17 µg/mL (Figure 12B). However, a decrease in nitric oxide production was observed after treatment with hydroxychavicol, compared with the control. At 20.78 µg/mL (LC5) and 21.57 µg/mL (LC10) of hydroxychavicol, the levels of nitric oxide production were 6.15 µM and 5.01 µM, respectively (Figure 12B). In addition, aspirin, a positive control, showed a nitric oxide production level of 2.35 µM in RAW 264.7 cells induced by LPS. 

## 3. Discussion

Poultry infection caused by antibiotic-resistant bacteria has become a serious issue worldwide. APEC is a significant bacterial pathogen that causes considerable economic losses in global poultry production, and it is considered a zoonotic pathogen [21]. Furthermore, the increasing prevalence of antibiotic resistance in bacteria makes treating these infections more challenging [22]. The present study showed the antibacterial activity of the nanoemulsion formulation containing *P. betle* leaves and hydroxychavicol, the pure compound presented in the leaves, against clinical isolates of APEC. It has been accepted that *P. betle* ethanol extract exhibits antibacterial activity against pathogens with MIC and MBC values ranging from 0.5 to 1.0 mg/mL [11]. Recently, our research group reported and confirmed the antibacterial activity of the ethanol *P. betle* extract against clinical isolates of APEC [11], *Salmonella* spp. [23], *Burkholderia pseudomallei* [19] and staphylococci associated with bovine mastitis [24]. However, the plant extract has limited solubility in water, making it difficult to apply in food or medicine. The development of nanoemulsion containing *P. betle* is a strategy that enhances the solubility of the extract. Our findings demonstrated that NEPE and hydroxychavicol exhibited strong antibacterial activity against APEC, with the potential to serve as effective antimicrobial agents. In the formulation, Tween 80 (T-80) and Tween 20 (T-20) were used as surfactants, while PG acted as a co-surfactant. Tween 80 and Tween 20 are widely used surfactants in emulsification and delivery systems. Tween 80, characterized by a longer unsaturated oleate hydrophobic tail, exhibits stronger surface activity and a greater ability to reduce interfacial tension compared to Tween 20, which has a shorter laurate tail. This structural difference contributes to the formation of larger and more stable nanoemulsion regions, enhancing overall emulsion stability [22]. It is accepted that surfactants play a crucial role in stabilizing nanoemulsions by reducing surface tension, improving solubility. Tween 80, with a higher hydrophilic–lipophilic balance (HLB), is commonly used to create stable emulsions and enhance the dispersion of hydrophobic compounds. Tween 20, which has a lower HLB, helps balance the system by stabilizing the oil and water phases. The inclusion of lactic acid and menthol further enhanced the solubility and antibacterial activity of the nanoemulsion. Smaller globule size leads to improved stability and bioavailability of active compounds [25]. Nanoemulsion systems formulated with hydrophobic deep eutectic solvents (HDESs), when combined with appropriate surfactants, are stable, increasing the solubility of hydrophobic actives and facilitating their absorption across biological membranes. Hydroxychavicol is characterized as a hydrophobic compound, as evidenced by its extraction and partitioning in an ethyl acetate solvent rather than in an aqueous phase [26,27]. Following the preparation of the formulations, *P. betle* leaf powder was incorporated, suspended, and subjected to extraction. The supernatant (extract) was collected and analyzed for its soluble hydroxychavicol content. It is posited that hydroxychavicol dissolves in the hydrophobic DES oil phase and the Tween 80/propylene glycol co-surfactant mixture. Our formulation and HPLC analysis substantiate the presence of the active compounds in a dissolved state. Nonetheless, further investigation into the solubility of hydroxychavicol within the specified components of the nanoemulsion is warranted. Small droplets provide a larger surface area, which may enhance the interaction between *P. betle* and bacterial cells, improving their antimicrobial and antibiofilm properties. The results of this study showed that Tween 80 played an important role in the formation of stable nanoemulsions. Both T-80-4 and T-80-7 formulations exhibited good characteristics, indicating uniform droplet distribution and good electrostatic stability. These results are consistent with the previous studies that developed nanoemulsions using Tween 80 as the primary surfactant and were able to produce particle sizes of 47.25 nm and PDI values of 0.21 [28] without phase separation even under accelerated stability tests. PDI values with relatively narrow particle size distributions are supported by the literature, where PDI values below 0.5 are generally considered indicative of narrow and uniform particle size distributions. Similarly, studies on polymer latexes and nanoparticle systems report that PDIs less than 0.3 to 0.5 correspond to narrow distributions, reflecting homogeneity and stability in particle size [29]. Overall, the use of Tween 80 not only contributed to smaller and more uniform droplet sizes but also improved the electrostatic stability of the system through a higher negative zeta potential. However, the substances used in the preparation of the emulsion possessed antibacterial activity. Lactic acid has been reported to inhibit the growth of *Erwinia persicina*, *Pseudomonas putida*, *Pseudomonas punonensis*, and *Citrobacter freundii*, with MIC values ranging from 1.56 to 3.12 mg/mL [30]. Therefore, the antibacterial activity of the emulsion containing *P. betle* was presented as a synergistic effect of the plant material and the chemical substances.

Phytochemical analysis by our research team revealed that hydroxychavicol and eugenol are the primary constituents in the ethanol extract of *P. betle* leaves [31]. Among the nanoemulsion formulations with HPLC analysis, hydroxychavicol was detected at high concentrations, while eugenol was present in lower amounts [32]. Notably, hydroxychavicol has been shown to disrupt bacterial cell division, leading to elongated bacterial cells without septum formation. The results demonstrated that APEC treated with hydroxychavicol exhibited elongated cell morphology without a septum. The effects of *P. betle* extract on APEC cells showed that the extract observed long cell formation and a dried shape [11]. This effect is likely due to hydroxychavicol, active compounds in *P. betle* extract, which may inhibit key division proteins. These results were supported by confocal microscope images, which showed that APEC cells treated with hydroxychavicol had an elongated shape, indicating that the cells were dead.

According to in silico studies, hydroxychavicol may affect the cell division protein of APEC due to the interaction of the compound and the cell division protein, as detected by molecular docking and dynamics simulation. This binding energy shows how strong and stable the molecular interaction is, which is why the five proteins with the highest binding energy (-ΔG), including ZapE, FtsW, FtsX, FtsZ, and FtsA, were chosen. It has been reported that the low binding energy values indicate stronger and more stable interactions between the ligand and the target protein [19]. Recently, molecular docking of berberine as an inhibitor of the FtsZ protein in *E. coli*. revealed the strong binding affinity of ZINC000524729297 (−8.73 kcal/mol) and ZINC000604405393 (−8.55 kcal/mol). Subsequently, the study revealed that the FtsZ-ZINC524729297 and FtsZ-ZINC000604405393 complexes had the lowest root-mean-square deviation, with the lowest binding energy and enhanced conformational stability in a dynamic environment [33]. The interaction between hydroxychavicol and selected cell division proteins was further confirmed by dynamic simulation. The FtsA protein showed the most stable conformation throughout the simulation, compared with other proteins. The RMSD values of the ligand reflect hydroxychavicol’s flexibility, suggesting that it may adapt to the binding site and form transient interactions without disrupting the protein’s natural structure, an important trait of effective molecular recognition [34]. The results showed that hydroxychavicol has a high ligand RMSD, indicating that it is more flexible and may adjust its shape while binding. Despite this flexibility, it still helps stabilize the overall structure of the proteins [35]. Hydroxychavicol’s stable binding to FtsA suggests that it disrupts bacterial cell division by interfering with the formation of division sites, which is crucial for *E. coli* viability. It has been reported that the FtsA mutation in *E. coli* results in the inhibition of φX174 protein-E, which mediates cell lysis [36].

We examined the cytotoxic effects of hydroxychavicol using RAW 264.7 cells to evaluate the safety of hydroxychavicol during the treatment of APEC infections. It was observed that hydroxychavicol at a concentration less than 12.5 µg/mL showed no signs of cytotoxicity after 24 h. Recently, the cytotoxic effects of hydroxychavicol against RAW 264.7 cells, as detected by MTT assay, were observed at 50 μM [37]. The investigation of cytotoxic effects of antimicrobial compounds on the cell lines is a preliminary test to evaluate the safety of the compounds. However, further work is required to determine the cytotoxicity of hydroxychavicol in other models such as animal models.

In this study, hydroxychavicol demonstrated effective anti-inflammatory effects by significantly suppressing nitric oxide production in LPS-induced RAW 264.7 cells. This finding suggests that hydroxychavicol may help reduce inflammation, which supports its traditional use in herbal medicine for treating infection-associated inflammatory conditions. Previous studies have found that hydroxychavicol led to a marked decrease in pro-inflammatory cytokines (IL-1β, TNF-α, and IL-6), nitric oxide levels, and lipid peroxidation, reflecting potent anti-inflammatory and antioxidant properties [38]. In addition, hydroxychavicol derivatives downregulate key inflammatory mediators and may exert an anti-inflammatory effect by inhibiting the NF-κB signaling pathway [39]. Hydroxychavicol exhibits strong potential as both an anti-bacterial and anti-inflammatory agent. Hydroxychavicol, with its combined antibacterial and anti-inflammatory properties, represents a promising candidate for developing new treatments. Its dual action offers potential benefits in combating infections while reducing inflammation, making hydroxychavicol-based therapies a novel and effective option for managing APEC infections and related inflammatory conditions. These findings highlight its promise as a therapeutic candidate for treating APEC infection and support the development of products that can inhibit APEC.

## 4. Materials and Methods

### 4.1. Bacterial Strains and Growth Conditions

The clinical isolates of APEC included CHUL7, CHUL8, CHUL9, CHUL10, CHUL13, CHUL47, CHUL49, CHUL50, CHUL53, and CHUL57. The bacteria were isolated from commercial broilers and native chickens and classified as APEC by our research team [40]. In addition, *E. coli* ATCC25922 was used as a reference strain. For bacterial growth conditions, all the bacteria were cultured on tryptic soy agar (TSA) and incubated at 37 °C for 24 h. The bacteria were then cultured in Tryptic soy broth (Difco, Claix, France), incubated at 37 °C for 24 h. The bacteria were kept at −80 °C in 20% glycerol until used.

### 4.2. Preparation of Plant Extract and Antimicrobial Compounds

#### 4.2.1. Preparation of Ethanol *P. betle* Extract

Fifty grams of dried *P. betle* leaf powder, collected from Phatthalung province, Thailand, was extracted in 200 mL of 95% ethanol for seven days at room temperature using a modified protocol described by [41]. The extract was then filtered and concentrated under reduced pressure. To ensure complete solvent removal, the extract was air-dried at room temperature until a stable weight was achieved through daily balancing. Then, the ethanol *P. betle* extract was dissolved in 100% dimethyl sulfoxide (DMSO) and stored at 4 °C until used.

#### 4.2.2. Preparation of HDES-Based Nanoemulsion Containing *P. betle* Leaf

Pseudo-ternary phase diagrams were constructed to identify the emulsion regions for HDES-based systems using lactic acid and menthol (1:2 molar ratio) as the oil phase, with surfactant mixtures of Tween 80:propylene glycol (PG) (1:1 *w/w*) or Tween 20:PG (1:1 *w/w*). The diagrams were generated by mixing varying weight ratios of oil and surfactant (10:0 to 0:10), followed by the gradual addition of water under constant stirring at 28 °C until homogeneity was achieved. These results were mapped using CHEMIX School software (Arne Standnes, Bergen, Norway).

The preparation of NEPEs was carried out using different component ratios (Table 1) and further extracted by microwave extraction. For the formulation labeled T-80-4, an initial mixture of lactic acid and menthol in a 1:2 ratio was created. Subsequently, Tween 80 and PG were combined in a 1:1 ratio. We utilized 30% lactic acid:menthol (1:2), 60% Tween 80:PG (1:1), and 10% water. The solutions were sonicated at a frequency of 37 kHz for 10 min. Following the preparation of these formulations, 10 g of *P. betle* leaf powder was added to each mixture and thoroughly mixed to ensure complete interaction of the powder. Then, the samples were extracted using a microwave with the conditions as follows: an electric power of 1000 watts for 10 s, repeated 3 times. The samples were centrifuged at 10,000× *g* for 10 min. The supernatant was collected and kept at 4 °C until used. The NEPE samples were analyzed using a Zetasizer Nano ZS (Malvern Panalytical, Malvern, UK) to determine their zeta potential and polydispersity index (PDI). For zeta potential measurements, the diluted samples were loaded into a capillary cell and analyzed at a scattering angle of 173°. PDI measurements were conducted using a quartz cuvette. Multiple runs (ranging from 10 to 100) were performed automatically, applying water’s refractive index of 1.33 and utilizing the Smoluchowski approximation for data interpretation. 

### 4.3. Detection of Hydroxychavicol in the NEPE

To detect hydroxychavicol in the NEPE using High-Performance Liquid Chromatography combined with Diode Array Detection (HPLC-DAD), hydroxychavicol standard solutions were prepared in a water–acetonitrile mixture (50:50, *v/v*) to generate a calibration curve. The NEPE was obtained through extraction methods, filtered, and diluted similarly. The HPLC analysis employed a vertical C15 column (5 μm particle size, 150 mm × 4.6 mm I.D.), with the diode array detector set at 280 nm. Gradient elution was used for the mobile gradient phase, starting with 50:50% (*v/v*) water and acetonitrile for 5 min, increasing acetonitrile to 80% (*v/v*) from 5 to 7 min, and maintaining 80% (*v/v*) acetonitrile until 12 min, with a flow rate of 1.0 mL/min and an injection volume of 20 µL. The hydroxychavicol standard solutions were injected to establish a calibration curve by plotting peak areas against concentrations. Following this, the NEPE was injected, and the hydroxychavicol peak area was recorded and compared to the calibration curve to quantify the hydroxychavicol content in the extract.

### 4.4. Determination of Minimal Inhibitory Concentration (MIC) and Minimal Bactericidal Concentration (MBC)

The determination of the MIC and MBC values of all antimicrobial agents against APEC was carried out using a broth microdilution assay as previously described [42]. The experiment was performed in triplicate in a 96-well microtiter plate. The ethanol extract and hydroxychavicol were serially diluted in Mueller Hinton broth (MHB) to give final concentrations as 2.0–0.0625 mg/mL. The concentrations of nanoemulsion and gentamicin (a positive control) were prepared as 1.0–0.03% *v/v* and 4.0–0.125 µg/mL, respectively. The bacterial suspension (100 μL, 1 × 10^6^ CFU/mL) was inoculated to each well and incubated at 37 °C for 18 h. The *E. coli* ATCC25922 strain was included as the reference strain. After incubation, the MIC values were determined using 0.03% of resazurin. The MIC was defined as the lowest concentration that inhibited bacterial growth and presented as a blue color. The MBC was further carried out from all wells that presented a blue color by streaking on TSA. The MBC was defined as the lowest concentration that killed the bacteria.

### 4.5. Time–Kill Study

Time–kill kinetic assay was used to confirm the antimicrobial effectiveness of NEPE and hydroxychavicol against a representative isolate of APEC. Bacterial cultures with an inoculum size of 5 × 10^5^ CFU/mL were grown in a medium containing NEPE and hydroxychavicol at concentrations equal to 1, 2, and 4 × MIC and incubated at 37 °C. DMSO (1%) was used as a negative control. Samples were taken at various intervals: 0, 2, 4, 6, 8, 12, 18, and 24 h. At each time point, 100 μL of the sample was serially diluted in sterile phosphate-buffered saline (PBS), and viable bacterial counts were determined using the drop plate method on TSA plates, which were incubated at 37 °C for 24 h. The experiment was repeated three times, and the results are presented as the mean log number of organisms ± standard deviation.

### 4.6. Evaluation of APEC Viability After Treatment with NEPE and Hydroxychavicol Using Confocal Microscopy

The viability of APEC after treatment with NEPE and hydroxychavicol was observed using a confocal microscope as previously described [43]. Briefly, the bacterial cells (1 × 10^6^ CFU/mL) were grown in TSB containing NEPE and/or hydroxychavicol at 1 × MIC and incubated at 37 °C for 18 h. The samples were centrifuged at 4000 rpm for 5 min, and the pellets were then washed twice with 0.01 M PBS solution. The pellets were re-centrifuged at 4000 rpm for 5 min and re-suspended in 100 μL Acridine orange (AO) and Propidium iodide (PI) staining solution, which was prepared by adding 200 μL of AO (0.5 mg/mL) and 200 μL PI (0.2 mg/mL) in 600 μL of 0.01 M PBS solution. The cell suspension was then incubated for 10 min at room temperature in the dark since both light-sensitive dyes were placed onto a slide and carefully covered with a coverslip. The cells were visualized using a fluorescent microscope. It was noticed that live cells turned green (AO), whereas the dead cells appeared red (PI).

### 4.7. Morphology of APEC After Treatment with NEPE and Hydroxychavicol Assessed Using Scanning Electron Microscopy (SEM)

The bacterial cells were cultured in TSB and incubated at 37 °C for 18 h. The bacterial cells (1 × 10^6^ CFU/mL) were then grown in a centrifuge tube containing NEPE and hydroxychavicol at 2 × MIC and 4 × MIC, incubated at 37 °C for 24 h. The final concentration of DMSO was 1%, and this concentration of DMSO was used as a negative control. The samples were then centrifuged at 5000 rpm for 5 min. The bacterial pellet was dropped on a sterile glass coverslip (0.5 cm × 0.5 cm) and air-dried. Specimens were fixed in 2.5% glutaraldehyde for 2 h. Then, the samples were dehydrated with a series of graded ethanol (20–100%) for 30 min at each step. After being mounted on aluminum stubs, the samples were dried using a critical point dryer. After that, the samples were covered in gold particles. The bacterial morphological characteristics such as their size, shape, and structure were examined under a scanning electron microscope.

### 4.8. Molecular Docking and Molecular Dynamic Simulation

The 3D structures of the selected protein [44] (FtsW—UniProt accession number: A0A0H2YVF7, FtsA—UniProt accession number: A0A0H2YVM0, FtsZ—UniProt accession number: A0A0H2YVY5, ZapC—UniProt accession number: A0A0H2YYA6, FtsP—UniProt accession number: A0A0H2Z2X2, FtsX—UniProt accession number: A0A0H2Z3S3, ZapE—UniProt accession number: A0A0H2Z455, ZapD—UniProt accession number: A1A7E5, FtsY—UniProt accession number: A0A0H2Z424 and ZipA—UniProt accession number: A1ADS9) were obtained from UniProt (https://www.uniprot.org), and were used as receptors for docking analysis. The chemical structure of hydroxychavicol (PubChem CID: 70775) was used as a ligand, which was sourced from PubChem (https://pubchem.ncbi.nlm.nih.gov). In addition, cefepime [45] was included as a positive control for the molecular docking assay. Both receptors and ligand were prepared and docked using Autodock Vina under Galaxy Version 1.5.7 (10.1002/jcc.21334). Docking conditions were performed in a grid box of 100 × 100 × 100. Results were analyzed based on binding scores or binding energy values to evaluate the interactions between hydroxychavicol and the target proteins. Molecular dynamics simulations were analyzed using tolls in Galaxy Europe Server (https://usegalaxy.eu, accessed on 1 April 2025). Five of ten proteins that were revealed to have low binding energy value were selected for further molecular dynamic simulations. Docked ligand (hydroxychavicol) and receptors (selected proteins) were cleaned using PyMOL software (version 4.60.)by removing water molecules and heteroatoms and adding hydrogen atoms. Cleaned ligand and receptors were run to establish the GROMACS topology (TOP) and position restraint (itp) under the SPC water model and the OPLS/AA force field. Then, protein–ligand complexes were run for TOP and itp that were used for configuration in a triclinic box (1 nm dimensions). The configurated complexes were run in SPC (generic three-point model) for solvation with a neutralized system. The solvated complexes were an additional run for energy minimization with the steepest descent algorithm and Fast Smooth Particle-Mesh Ewald (SPME) electrostatics (5 × 10^5^ of number of steps, 10^4^ of EM tolerance, and 10^−2^ nm of maximum step size). The obtained potential energy (kJ/mol) and number of steps during the energy minimization were plotted. Then, the equilibrations under an isothermal–isochoric (NVT) ensemble and an isothermal–isobaric (NPT) ensemble were performed. For the NVT, the leap-frog algorithm for integrating Newton’s equations of motion was selected under 300 K, 0.002 step length in ps, 10^4^ of the number of steps that elapse between saving data points, and 5 × 10^6^ of the number of steps for the simulation. In addition to the NPT, the leap-frog algorithm for integrating Newton’s equations of motion was also performed under the same conditions as the NVT. Then, the main simulation was conducted under 300 K, with a time step of 0.001, data saved every 5 × 10^3^ steps, and a total of 5 × 10^6^ simulation steps. All simulation outputs were analyzed using structure and trajectory files, which were performed using software in Galaxy tools (25.0.2.dev0 version). The root mean square deviation (RMSD) and root mean square fluctuation (RMSF) were performed for protein and hydroxychavicol conformation stability. In addition, principal component analysis (PCA) and hydrogen bond analyses were additionally analyzed.

### 4.9. Cytotoxicity Assay

RAW 264.7 cells were cultured in Dulbecco’s Modified Eagle’s Medium (DMEM; Gibco, Thermo Fisher Scientific, Bohemia, NY, USA) supplemented with 10% fetal bovine serum (Gibco, Bohemia, NY, USA) and 1% penicillin–streptomycin solution (Gibco, Thermo Fisher Scientific), incubated at 37 °C in 5% CO_2_. The cells were subcultured and plated at 80–90% confluency using a sterile cell scraper. The cells were seeded into 96-well plates at a density of 6 × 10^5^ cells/well and allowed to adhere overnight.

Cytotoxic effects of the hydroxychavicol on RAW 264.7 cells were investigated by MTT assay using a modified protocol described by [46]. The stock solution of hydroxychavicol was diluted in DEME to achieve a final concentration (1.56–25 µg/mL) in each well of 100 μg/mL. After incubation for 24 h, the cells were treated with 100 μL of fresh media along with 10 μL of 3-(4,5-dimethylthiazolyl)-2,5-diphenyltetrazolium bromide (MTT) solution (5 mg/mL) and incubated at 37 °C for 4 h. The medium containing MTT was removed and 200 μL of DMSO was added. The absorbance was determined by a microplate reader at a wavelength of 570 nm. The percentage of cell viability was calculated and compared to a negative control. The experiment was repeated in triplicate three times to ensure reproducibility. The percentage of cell viability was calculated based on optical density (OD) values obtained from the MTT (or relevant cytotoxicity) assay using the following formula:

% cell viability = (OD of test/OD of untreated control) × 100

### 4.10. Measurement of Nitric Oxide

The RAW 264.7 cells were seeded at a density of 6 × 10^5^ cells/well in 96-well plates and incubated at 37 °C for 24 h under 5% CO_2_. Subsequently, the media were removed and fresh FBS-free DMEM media were replaced [47]. Effects of hydroxychavicol on nitric oxide production in RAW 264.7 were assessed at concentrations of LC_5_ (20.78 µg/mL) and LC_10_ (21.57 µg/mL). The cells were then incubated at 37 °C for 24 h under 5% CO_2_. After 1 h of the treatment, cells were stimulated with 1 μg/mL of LPS and incubated at 37 °C for 24 h under 5% CO_2_. The nitric oxide production was measured by treating the supernatant with an equal volume of Griess reagent (Sigma-Aldrich, St. Louis, MO, USA). Then, the absorbance was measured at 540 nm in a microplate reader. Each test was performed in triplicate. The amount of nitrite in the culture media was determined using a standard curve constructed from different concentrations of sodium nitrite (NaNO_2_). The absorbance was measured at 540 nm following the Griess reaction. The concentration of nitric oxide (NO) production in the test samples was then calculated using the following equation:

Nitric oxide production = (OD of test/OD of standard) × concentration of standard

### 4.11. Statistical Analysis

All experiments were performed in triplicate, and the recorded data were analyzed using descriptive statistics, e.g., mean and standard deviation (S.D.). One-way ANOVA was selected to test the difference among the experimental groups. The significance of the one-way ANOVA was further tested using Duncan’s test. All statistical analyses were performed under a 95% confidence interval, and a *p*-value < 0.05 was considered for statistical significance. 

## 5. Conclusions

This study revealed the antibacterial effectiveness of NEPE and hydroxychavicol against APEC. SEM analysis demonstrated that APEC cells treated with hydroxychavicol exhibited filamentous cells with incomplete septa. As a key target, molecular docking and dynamics simulations implicated FtsA, a divisome protein critical for Z-ring assembly. Hydroxychavicol formed stable complexes with FtsA, likely impairing its membrane-anchoring function. The results suggest that NEPE and hydroxychavicol may have promising antibacterial potential for inhibiting APEC growth.

## 6. Patents

This research received petty patent number 2503001466 for the “Formulation and extraction process of hydroxychavicol from *Piper betle* leaves in microemulsion form”.

## Figures and Tables

**Table 1:** Different formulations of the hydrophobic deep eutectic solvent-based nanoemulsion containing *P. betle* leaf phytochemicals (NEPE).

Formulations of NEPE	Ingredients (g)			
	Lactic Acid: Menthol (1:2 Mole Ratio)	Surfactant Mixture	Water	*P. betle* Powder
T-80-4	30	60 ^a^	10	10
T-80-7	40	50 ^a^	10	10
T-20-4	30	60 ^b^	10	10
T-20-7	40	50 ^b^	10	10

^a^ Surfactant mixtures are the mixture of Tween 80 and propylene glycol at a 1:1 weight ratio. ^b^ Surfactant mixtures are the mixture of Tween 20 and propylene glycol at a 1:1 weight ratio.

**Table 2:** Contents of hydroxychavicol in the hydrophobic deep eutectic solvent-based nanoemulsion containing *P. betle* leaf phytochemicals (NEPE).

Formulations of NEPE	Characteristics			
	Hydroxychavicol Detected (mg/mL)	Particle Size (nm)	Polydispersity Index (PDI)	Zeta Potential (mV)
T-80-4	4.24 ± 0.01 ^a^	162.60 ± 4.255 ^a^	0.46 ± 0.01 ^a^	−45.53 ± 0.17 ^a^
T-80-7	2.62 ± 0.01 ^b^	353.30 ± 26.535 ^a^	0.31 ± 0.04 ^ab^	−47.63 ± 0.09 ^b^
T-20-4	2.20 ± 0.01 ^c^	642.33 ± 121.64 ^a^	0.62 ± 0.08 ^c^	−23.97 ± 0.81 ^c^
T-20-7	1.88 ± 0.01 ^d^	24,693.33 ± 899.79 ^b^	0.61 ± 0.09 ^bc^	−16.03 ± 0.53 ^d^

Different superscript letters (a, b, c, d) indicate significant differences (*p* < 0.05) according to Duncan’s test.

**Table 3:** The MIC and MBC values of the tested agents against clinical isolates of APEC.

Isolates	MIC/MBC						
	NEPE ^a^				Ethanol *P. betle* Extract ^b^	Hydroxychavicol ^b^	Gentamicin ^c^
	T-80-4	T-80-7	T-20-4	T-20-7			
CHUL7	0.06/0.125	0.06/0.125	0.125/0.25	0.06/0.125	1.0/1.0	0.25/0.25	4/4
CHUL8	0.06/0.125	0.06/0.25	0.125/0.25	0.06/0.25	1.0/1.0	0.25/0.25	1/1
CHUL9	0.06/0.25	0.125/0.125	0.125/0.25	0.125/0.125	1.0/1.0	0.25/0.25	0.5/1
CHUL10	0.06/0.25	0.125/0.125	0.125/0.25	0.125/0.125	1.0/1.0	0.25/0.25	1/2
CHUL13	0.125/0.25	0.125/0.25	0.125/0.25	0.125/0.125	1.0/1.0	0.25/0.25	0.5/1
CHUL47	0.25/0.25	0.125/0.125	0.25/0.25	0.125/0.125	1.0/1.0	0.25/0.25	0.5/2
CHUL49	0.25/0.25	0.125/0.25	0.125/0.25	0.125/0.125	1.0/1.0	0.25/0.25	0.5/2
CHUL50	0.125/0.125	0.125/0.125	0.125/0.25	0.125/0.25	1.0/1.0	0.25/0.25	1/2
CHUL53	0.125/0.25	0.125/0.125	0.125/0.125	0.125/0.125	1.0/1.0	0.25/0.25	1/2
CHUL57	0.125/0.125	0.125/0.125	0.06/0.125	0.125/0.125	1.0/1.0	0.25/0.25	0.5/1
ATCC25922	0.125/0.125	0.125/0.125	0.125/0.125	0.125/0.125	0.5/1.0	0.25/0.25	0.5/1

^a^ = % *v/v*; ^b^ = mg/mL; ^c^ = µg/mL.

**Table 4:** Comparison of binding energies between hydroxychavicol and cefepime for bacterial cell division proteins.

Protein	Binding Energy	
	Hydroxychavicol	Cefepime
ZapE	−5.891	−6.887
FtsW	−5.887	−6.575
FtsX	−5.642	−6.334
FtsZ	−5.416	−8.057
FtsA	−5.296	−6.897
ZapC	−5.034	−6.373
ZapD	−5.027	−6.116
FtsP	−5.002	−6.920
FtsY	−4.839	−5.676
ZipA	−4.706	−5.823

## Data Availability

The original contributions presented in this study are included in the article. Further inquiries can be directed to the corresponding author.

## Supplementary Materials

The following supporting information can be downloaded at: https://www.mdpi.com/article/10.3390/antibiotics14080788/s1, Table S1: The MIC and MBC values of the nanoemulsion base solution against clinical isolates of APEC.
