# Supplementary material for: Bactericidal Activities of Nanoemulsion Containing Piper betle L. Leaf and Hydroxychavicol Against Avian Pathogenic Escherichia coli and Modelling Simulation of Hydroxychavicol Against Bacterial Cell Division Proteins
Source: Antibiotics (Basel). 2025 Aug 3;14(8):788. doi: 10.3390/antibiotics14080788 (PMC12382705; doi:10.3390/antibiotics14080788)
Supplement: Supplementary file 1 [file antibiotics-14-00788-s001.zip › antibiotics-3720176-supplementary.pdf]

Table S1. The MIC and MBC values of the nanoemulsion base solution against clinical isolates of APEC

Isolates	MIC/MBC			
	Nanoemulsion base solution			
	T-80-4	T-80-7	T-20-4	T-20-7
CHUL7	0.5/>1.0	0.25/>1.0	0.5/>1.0	0.5/>1.0
CHUL8	1.0/>1.0	0.5/1.0	0.5/>1.0	1.0/1.0
CHUL9	0.5/>1.0	0.5/1.0	0.5/1.0	1.0/>1.0
CHUL10	0.25/>1.0	0.5/1.0	0.5/1.0	0.5/1.0
CHUL13	1.0/>1.0	1.0/>1.0	1.0/>1.0	1.0/>1.0
CHUL47	0.5/0.5	0.5/>1.0	0.5/1.0	0.5/1.0
CHUL49	1/>1.0	0.5/>1.0	1/>1.0	1/>1.0
CHUL50	0.25/>1.0	0.5/1.0	0.5/1.0	1.0/1.0
CHUL53	0.5/>1.0	0.25/1.0	0.5/>1.0	0.5/1.0
CHUL57	0.5/>1.0	0.25/>1.0	0.5/>1.0	0.5/>1.0
ATCC25922	0.25/>1.0	0.25/>1.0	0.25/>1.0	0.5/1.0
